# Metformin suppresses cancer initiation and progression in genetic mouse models of pancreatic cancer

**DOI:** 10.1186/s12943-017-0701-0

**Published:** 2017-07-24

**Authors:** Ke Chen, Weikun Qian, Zhengdong Jiang, Liang Cheng, Jie Li, Liankang Sun, Cancan Zhou, Luping Gao, Meng Lei, Bin Yan, Junyu Cao, Wanxing Duan, Qingyong Ma

**Affiliations:** Department of Hepatobiliary Surgery, First Affiliated Hospital, Xi’an Jiaotong University, 277 West Yanta Road, Xi’an, 710061 China

**Keywords:** Metformin, Pancreatic cancer, Tumorigenesis, Chronic pancreatitis

## Abstract

**Background:**

Pancreatic ductal adenocarcinoma (PDAC) is the fourth leading cause of cancer-associated mortality worldwide with an overall five-year survival rate less than 7%. Accumulating evidence has revealed the cancer preventive and therapeutic effects of metformin, one of the most widely prescribed medications for type 2 diabetes mellitus. However, its role in pancreatic cancer is not fully elucidated. Herein, we aimed to further study the preventive and therapeutic effects of metformin in genetically engineered mouse models of pancreatic cancer.

**Methods:**

LSL-Kras^G12D/+^; Pdx1-Cre (KC) mouse model was established to investigate the effect of metformin in pancreatic tumorigenesis suppression; LSL-Kras^G12D/+^; Trp53^fl/+^; Pdx1-Cre (KPC) mouse model was used to evaluate the therapeutic efficiency of metformin in PDAC. Chronic pancreatitis was induced in KC mice by peritoneal injection of cerulein.

**Results:**

Following metformin treatment, pancreatic acinar-to-ductal metaplasia (ADM) and mouse pancreatic intraepithelial neoplasia (mPanIN) were decreased in KC mice. Chronic pancreatitis induced a stroma-rich and duct-like structure and increased the formation of ADM and mPanIN lesions, in line with an increased cytokeratin 19 (CK19)-stained area. Metformin treatment diminished chronic pancreatitis-mediated ADM and mPanIN formation. In addition, it alleviated the percent area of Masson’s trichrome staining, and decreased the number of Ki67-positive cells. In KPC mice, metformin inhibited tumor growth and the incidence of abdominal invasion. More importantly, it prolonged the overall survival.

**Conclusions:**

Metformin inhibited pancreatic cancer initiation, suppressed chronic pancreatitis-induced tumorigenesis, and showed promising therapeutic effect in PDAC.

**Electronic supplementary material:**

The online version of this article (doi:10.1186/s12943-017-0701-0) contains supplementary material, which is available to authorized users.

## Background

Pancreatic ductal adenocarcinoma (PDAC) is the fourth leading cause of cancer-associated mortality worldwide with a mortality that closely parallels incidence. Although great efforts have been made, the 5-year survival of PDAC is still less than 7% [1]. Most patients remain asymptomatic until they develop to an advanced stage with complications involving distant metastasis. Surgical resection is regarded as the only potentially curative treatment. Gemcitabine, S-1, or an oral fluoropyrimidine derivative is given as adjuvant chemotherapy for surgery. For those who are not eligible for surgical resection but have good performance status, FOLFIRINOX (fluorouracil, folinic acid [leucovorin], irinotecan, and oxaliplatin) and gemcitabine plus nanoparticle albumin-bound paclitaxel (nab-paclitaxel) are regarded as the treatments of choice [2]. However, even for those who received a complete surgical resection, 5-year survival is still approximately 25% [2, 3].

PDAC mostly arises from microscopic noninvasive precursor lesions. Based on the pathological architecture and the degree of cytological atypia, precursor lesions are graded into several grades, including acinar-to-ductal metaplasia (ADM) and pancreatic intraepithelial neoplasia (PanIN), which is further divided into three grades, namely PanIN1, PanIN2 and PanIN3 [4]. Desmoplastic reaction, a dominant character of PDAC, is formed by the activation of pancreatic stellate cells (PSCs), which synthesize and secrete large amounts of extracellular matrix (ECM) [5]. Kras is one of the most common genes mutated in PDAC and plays a crucial role in the initiation and progression of PDAC, in which Kras mutation occurs in more than 90% of patients [6]. Somatic mutations in the Trp53 tumor suppressor gene are another frequent genetic event that drives PDAC progression. Substantial evidence has identified various molecular mechanisms, including JAK/STAT3 signaling and epidermal growth factor receptor (EGFR) signaling, both of which are important factors proven to be required for Kras-induced tumorigenesis [7, 8].

Metformin is one of the most widely prescribed medications for type 2 diabetes mellitus. Substantial epidemiologic and clinical studies have suggested its cancer therapeutic potential [9]. However, its cancer preventive and therapeutic effects and the mechanisms involved in pancreatic cancer are not fully elucidated. A previous case-controlled study suggested that diabetic patients who had taken metformin showed a significantly lower risk of pancreatic cancer compared with those who had not taken metformin [10]. In addition, metformin use was associated with an improved outcome in diabetic patients with pancreatic cancer [11, 12]. Metformin inhibited pancreatic cancer cell and tumor growth by down-regulating Sp transcriptional factors and showed an impact on the tumor microenvironment in PDAC [13–15]. Our previous study revealed that in human PDAC tissue, AMPK inactivation is correlated with desmoplastic reaction and patients’ poor prognosis. Our subsequent in vitro study found that the activation of AMPK by metformin inhibits pancreatic cancer invasion and migration. In addition, metformin suppresses TGF-β-induced PSC activation. In accordance with in vitro findings, metformin reduced tumor growth and desmoplasia in subcutaneous and orthotopic models of PDAC [15].

In the current study, using an oncogenic Kras-mediated and cerulein-induced mouse model of chronic pancreatitis in LSL-Kras^G12D^; Pdx1-Cre (KC) mice, as well as LSL-Kras^G12D/+^; Trp53^fl/+^; Pdx1-Cre (KPC) mouse model, we aimed to further investigate the cancer preventive and therapeutic effects of metformin. Interestingly, we observed a delayed formation of precursor lesions and impaired tumor progression following metformin treatment.

## Methods

### Genetically engineered transgenic mice

Pdx1-Cre mice, LSL-Kras^G12D^ mice and Trp53^fl/fl^ mice were purchased from the Nanjing Biomedical Research Institute of Nanjing University, Nanjing, China. The breeding of LSL-Kras^G12D^; Pdx1-Cre (KC) transgenic mice was achieved by crossing LSL-Kras^G12D^ mice with Pdx1-Cre mice (Additional file 1: Fig. S2A). LSL-Kras^G12D/+^; Trp53^fl/+^; Pdx1-Cre (KPC) mice were obtained by firstly crossing Trp53^fl/fl^ mice with Pdx1-Cre mice to generate Trp53^fl/fl^; Pdx1-Cre offspring. Trp53^fl/fl^; Pdx1-Cre mice were then crossed with LSL-Kras^G12D^ mice to generate KPC animals (Additional file 1: Fig. S2B). Polymerase chain reaction (PCR) was applied for the genotyping of transgenic mice (Additional file 2: Fig. S3). The primer sequences used for the genotyping of transgenic mice were presented in Additional file 3: Table S1. All mice were housed under pathogen-free conditions and with free access to water and food. All experimental protocols were approved by the Ethical Committee of the First Affiliated Hospital of Medical College, Xi’an Jiaotong University, Xi’an, China.

### Induction of chronic pancreatitis

To induce chronic pancreatitis, cerulein (Sigma, St. Louis, MO, USA) was administered daily by intraperitoneal injection (0.1 ml of a 50 mg/ml solution in saline) 5 days per week as previously described [16]. Mice were treated for 4 consecutive weeks and allowed to recover for 1 week before harvesting the tissue.

### Tissue preparation and histology

Mice were sacrificed, and the pancreas and other organs such as the liver and lungs were gently removed. The pancreas tissues were weighed and the tumor volumes were measured; then, the tissues were immediately fixed in 10% buffered formalin and embedded in paraffin. For histopathological analysis, tissues were sliced (5 μm), and Hematoxylin & Eosin (H&E) staining was performed according to the manufacturer’s instructions. Identification of ADM and grading of mPanIN (graded as mPanIN1A, mPanIN1AB, mPanIN2, and mPanIN3) and PDAC were based on criteria described previously [17]. For quantification of ADM and mPanIN lesions, five 10X pictures were randomly taken in every section, and the numbers of ADM and mPanIN lesions were calculated. Liver and lung were serially sectioned, and every fifth section was stained with H&E for the recognition of distant metastasis.

### Immunohistochemistry

Immunohistochemical staining was performed using the SABC kit (Maxim, Fuzhou, China) according to the manufacturer’s instructions. Briefly, the pancreas tissue sections were incubated in primary antibodies for CK19 (Abcam, Cambridge, MA, USA), phospho-STAT3 (CST, Danvers, MA, USA), phospho-AMPK (CST, Danvers, MA, USA), phospho-mTOR (Abcam, Cambridge, MA, USA), and α-SMA (Abcam, Cambridge, MA, USA) overnight at 4 °C; then, sections were incubated in the appropriate biotinylated secondary antibody for 30 min at room temperature, followed by 30 min of incubation with streptavidin peroxidase (Dako LSAB + HRP kit). After rinsing, the results were visualized using DAB, and the slides were counterstained with hematoxylin.

### Masson’s trichrome staining

Trichrome staining was performed using the Sigma Trichrome Stain (Masson) Kit according to the manufacturer’s instructions. To quantitatively evaluate trichrome-stained fibers in each group, representative slides per mouse were chosen, and at least 5 10X pictures were taken by light microscopy from each slide; then, the percentages of stained area were calculated using Image J software.

### In vivo treatment with metformin

Metformin was administered at 200 mg/kg daily by gavage. According to the Reagan-Shaw method for dose translation from animal to human studies [18], the human equivalent of a murine dose of 200 mg/kg is 972 mg for an average sized 60 kg adult human. Therefore, the selected dose in the present study is within the safe therapeutic range reported in humans (1000 to 2500 mg).

### Statistical analysis

The data are presented as the mean ± SD. Comparisons between groups were analyzed by Student’s t-test. Kaplan-Meier analysis was used for survival analysis. *P* values <0.05 were considered significant.

## Results

### Genetically engineered mice recapitulated the histopathological characteristics of PDAC in human patients

**Fig. 1:**
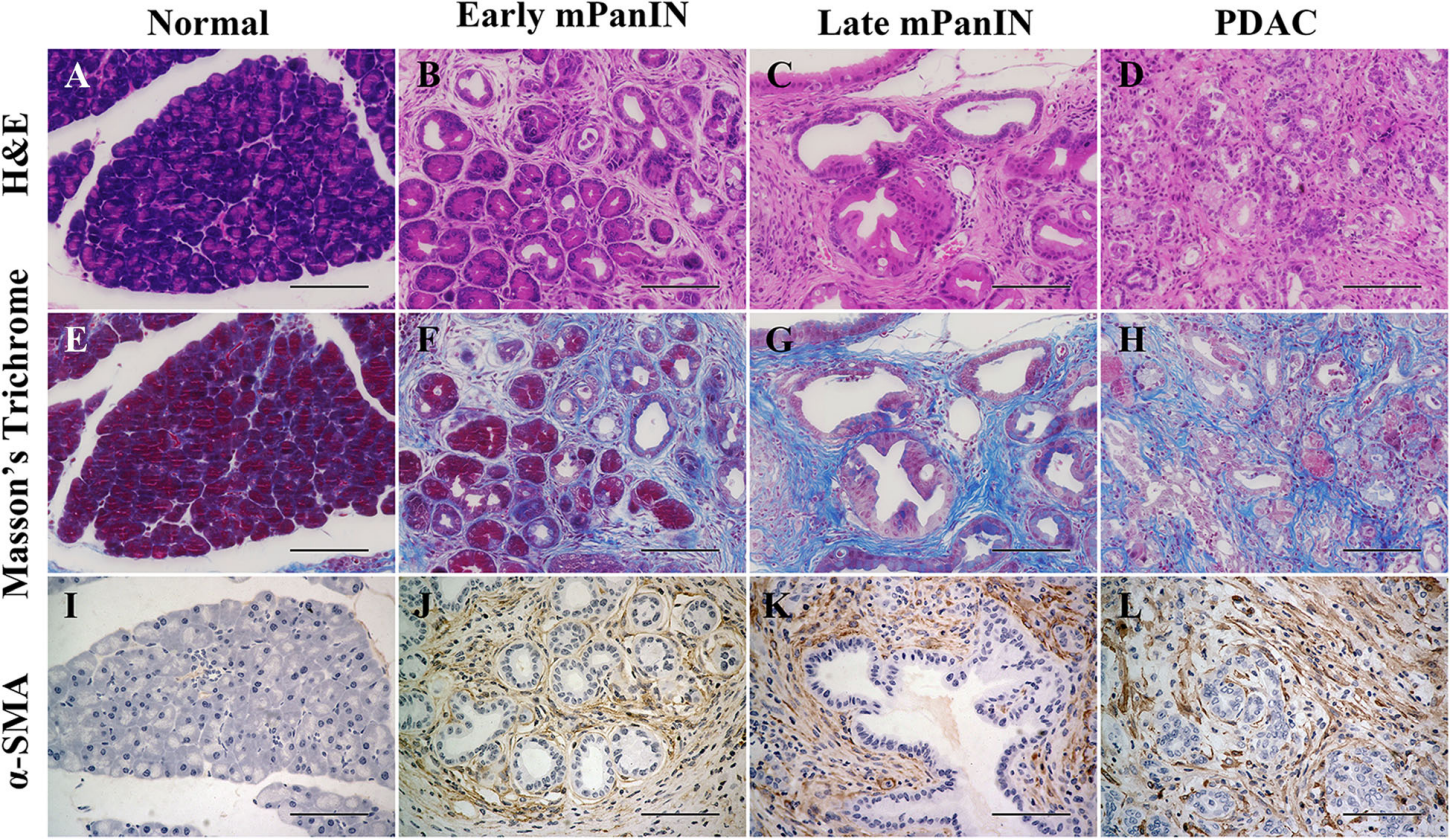
Genetically engineered mice recapitulate the pathological characteristics of pancreatic cancer. **a**-**d** Representative images of normal pancreas tissue (**a**), early mPanIN (**b**), late mPanIN (**c**) and invasive PDAC (**d**) stained by hematoxylin and eosin (H&E). **e**-**h** Masson’s trichrome staining of different stages of pancreatic precursor lesions and invasive PDAC. **i-h** Immunohistochemical staining of α-SMA in different stages of mouse pancreatic precursor lesions and PDAC. Scale bars =100 μm

Genetically engineered mouse models have the potential to assist our understanding of the histopathological characteristics of noninvasive and invasive pancreatic neoplasia and thus facilitate the development of preventative and therapeutic strategies for PDAC, as well as progress novel tests for the early detection of pancreatic neoplasia. Firstly, we sacrificed the KC and KPC mice at different time points to investigate the kinetics of tumor formation, as demonstrated in Additional file 4: Fig. S1. In line with previous reports [19, 20], we found that in early mPanIN lesions, the lesions presented as flat epithelial lesions composed of columnar cells with basally located nuclei and supranuclear mucin (Fig. 1b). In late mPanIN, the flat epithelium turned to papillary structures accompanied by nuclear abnormalities, including loss of polarity and nuclear crowding (Fig. 1c). PDAC in the transgenic engineered mice presented as chaos of the architecture and cancer cells interspersed in abundant stroma (Fig. 1d). Masson’s trichrome staining was performed to observe the desmoplastic reaction during PDAC progression. We found that Masson’s trichrome staining was detected in early precursor lesions such as ADM and mPanIN1 (Fig. 1f), and it was exacerbated when the lesions progressed to late mPanIN and invasive PDAC (Fig. 1g, h). Consistent with Masson’s trichrome, α-SMA staining was detected and showed an increasing tendency from early mPanIN to invasive PDAC (Fig. 1i-l). These results reinforced the resemblance of pancreatic cancer initiation and progression in genetically engineered mice and human patients.

### Metformin suppressed precursor lesion formation in KC mice

**Fig. 2:**
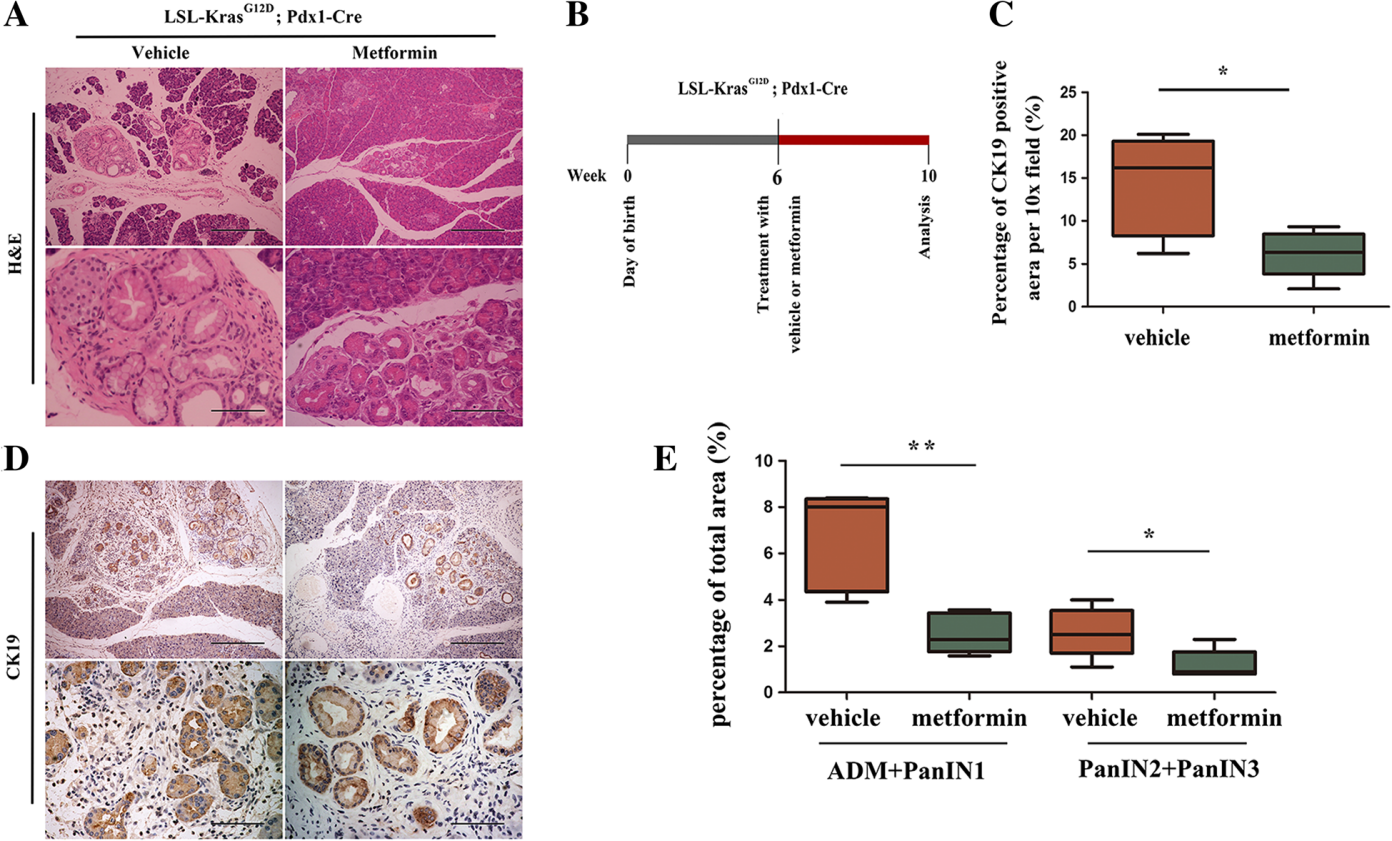
Metformin impaired oncogenic Kras-mediated mPanIN formation in KC mouse model. **a** H&E staining of the pancreas in vehicle or metformin-treated mice. Scale bars: (top row) = 400 μm; (2nd row) = 100 μm. **b** Scheme showing the experimental design of metformin treatment protocols in mice. **c** Quantification of the percentage of CK19 positive duct-like structures in mice treated with vehicle or metformin. **d** Representative images show pancreatic precursor lesions stained by anti-CK19. Scale bars: top row = 400 μm; 2nd row = 100 μm. **e** Quantification of the percentage of early mPanIN (ADM plus mPanIN1) and late mPanIN (mPanIN2 plus mPanIN3) in mice treated with vehicle or metformin. *P < 0.05; **P < 0.01

To test whether the intake of metformin was sufficient to suppress pancreatic preneoplastic lesion formation, we investigated the cancer preventative effect of metformin in KC mouse model. Starting at 6 weeks of age, KC mice were treated daily with metformin (200 mg/kg) or vehicle for 4 weeks by gavage (Fig. 2b). Then, the mice were sacrificed, and a histological examination was performed to evaluate the effect of metformin on ADM and mPanIN formation (Fig. 2a). Cytokeratin 19 (CK19) was stained to show the duct-like lesions (Fig. 2d). We showed that at the end time point, the pancreas from KC mice presented with multifocal lesions, which were composed of early precursor lesions (ADM and mPanIN1) and less late mPanIN lesions (mPanIN2 and mPanIN3). However, following metformin treatment, the percentage of early and late mPanIN lesions was decreased (Fig. 2e). We also detected a decreased percentage of CK19-stained area (Fig. 2c). We conclude that metformin suppressed initiation of pancreatic cancer in KC mice.

### Metformin suppressed chronic pancreatitis-induced pancreatic tumorigenesis

**Fig. 3:**
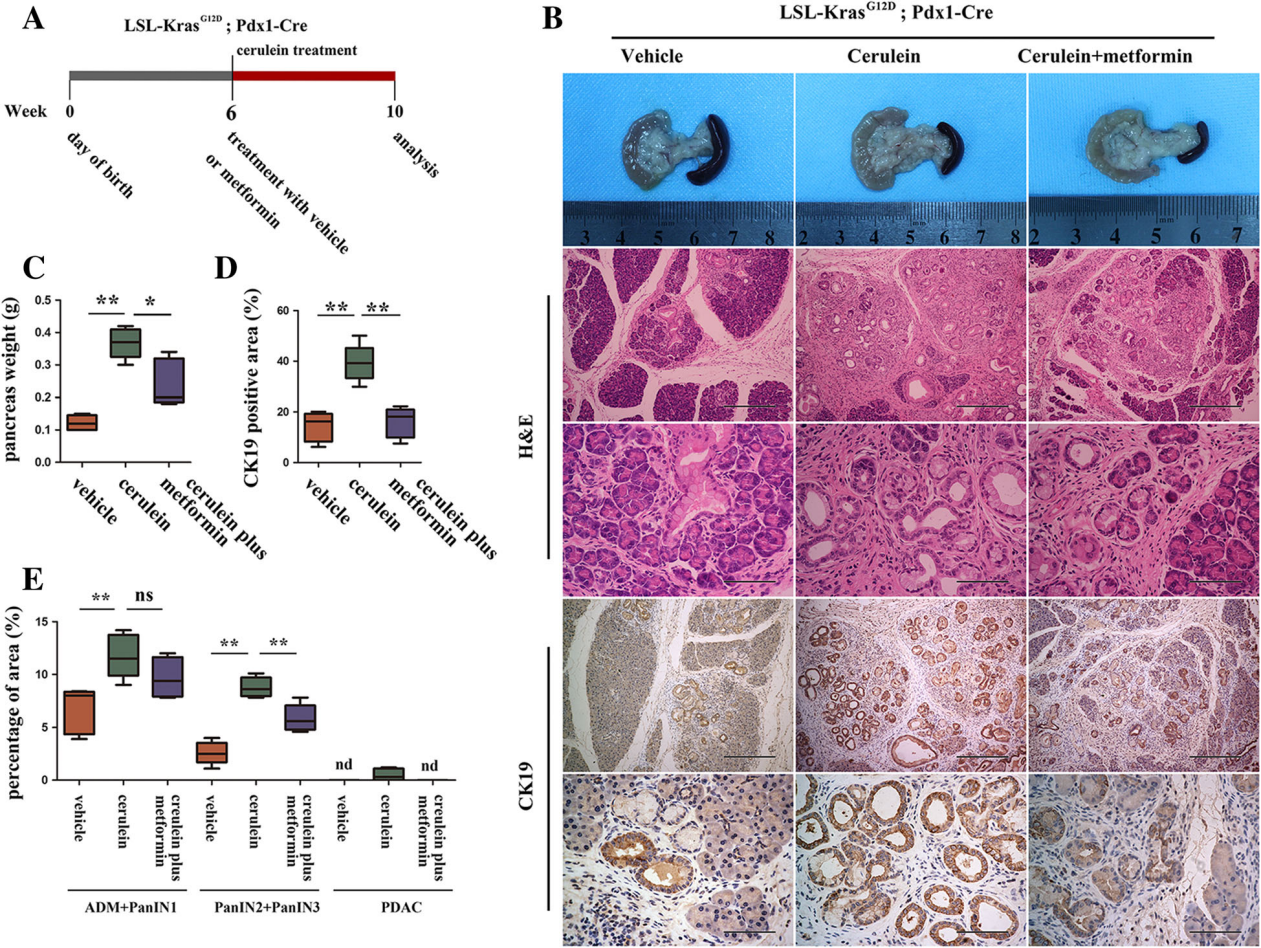
Metformin suppressed chronic pancreatitis-associated tumorigenesis. **a** Schematic presentation of the induction of chronic pancreatitis and treatment of metformin in KC mice. **b** Macroscopic picture, histology and IHC staining of CK19 in mice treated with vehicle, cerulein, or cerulein plus metformin. Scale bars: H&E (top row) = 400 μm; (2nd row) = 100 μm; CK19 (top row) = 400 μm; (2nd row) = 100 μm. **c** Quantification of the weight of the pancreas. **d** Statistical analysis of the CK19-positive area. (**e**) Quantification of the percentages of early pancreatic lesions (ADM plus mPanIN1), late mPanIN lesions (ADM plus PanIN1) and PDAC in mice treated with vehicle, cerulein, or cerulein plus metformin. nd, not detected. *P < 0.05; **P < 0.01

Chronic pancreatitis is a widely accepted risk factor for PDAC [21]. To investigate the role of metformin in chronic pancreatitis-associated tumorigenesis, KC mice were treated chronically with a low dose of cerulein (0.1 ml of a 50 μg/ml solution in saline), a cholecystokinin analog that can induce the secretion of pancreatic enzymes, 5 days per week [16]. The pancreas tissues of mice treated with cerulein or cerulein combined with metformin were analyzed. As expected, chronic treatment with cerulein induced a coarse and granular macroscopic appearance of the pancreas (Fig. 3b), in line with an increase in the weight of the pancreas tissue (Fig. 3c). The hematoxylin & eosin (H&E) staining of cerulein-treated pancreas showed a replacement of the normal pancreatic acini with glandular metaplasia and mPanIN lesions, which was accompanied by obvious fibrosis. We also observed frequent late mPanIN lesions and focused areas of PDAC (Fig. 3e). IHC showed significantly increased staining of CK19 in pancreatic tissues with chronic pancreatitis (Fig. 3b).

Surprisingly, compared to mice treated with cerulein alone, mice treated with metformin showed decreased pancreas weight (Fig. 3c). The histology showed large areas of normal acini preserved (Fig. 3b). Statistical analysis suggested that metformin treatment significantly suppressed the progression of precursor lesions, with a decreased percentage of mPanIN2 plus mPanIN3 and a decreased incidence of PDAC (Fig. 3e). Accordingly, the CK19-positive area was decreased (Fig. 3d). These results show that treatment with metformin delayed chronic pancreatitis-induced pancreatic oncogenesis.

### Metformin reduced fibrosis in mice with chronic pancreatitis.

**Fig. 4:**
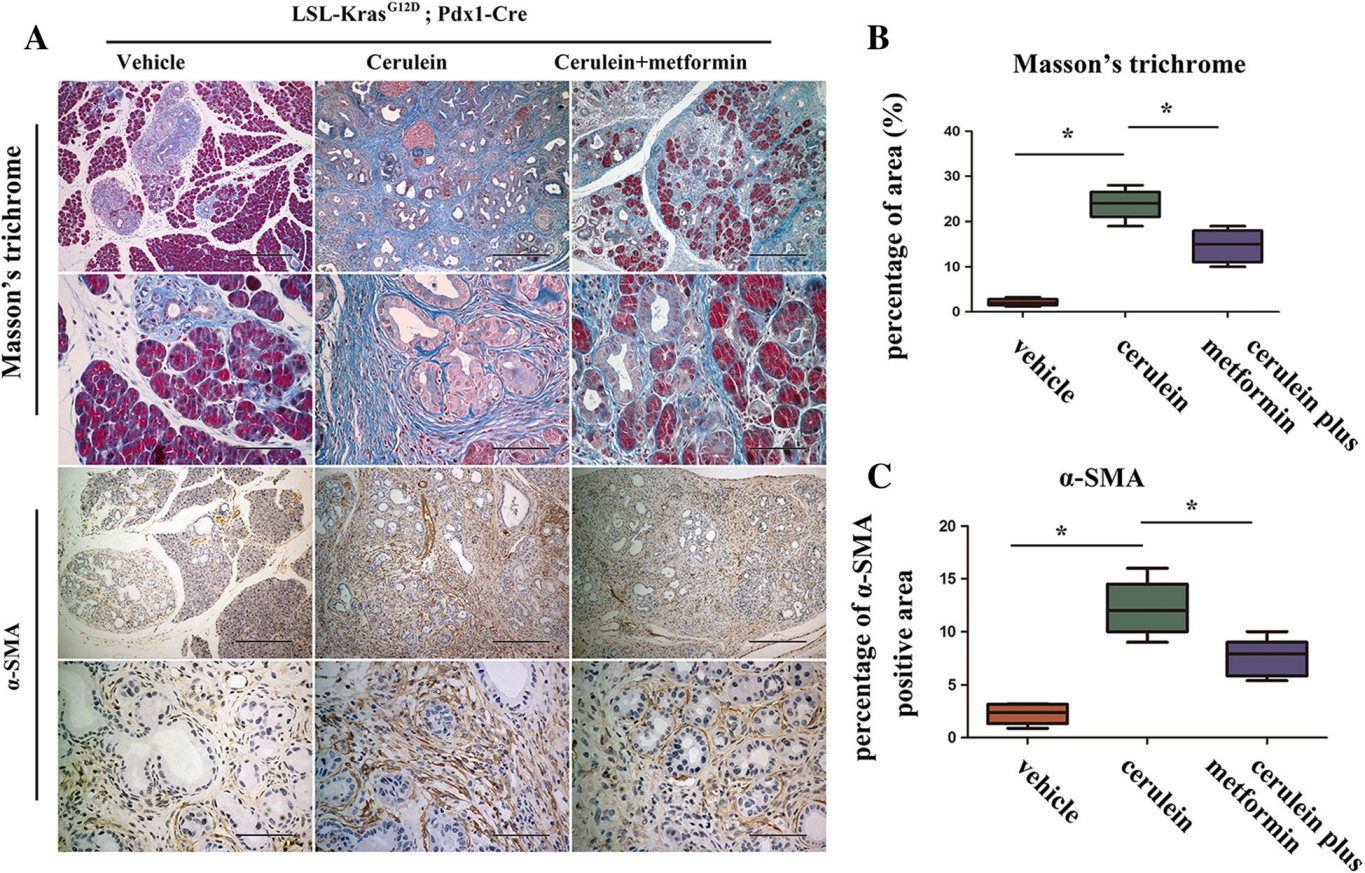
Chronic pancreatitis-induced pancreatic fibrosis was decreased following metformin treatment. **a** Masson’s trichrome staining and immunohistochemical staining of α-SMA. Scale bars: H&E (top row) = 400 μm; (2nd row) = 100 μm; α-SMA (top row) = 400 μm; (2nd row) = 100 μm. **b**-**c** Quantification of Masson’s trichrome and α-SMA in mice treated with vehicle, cerulein, cerulein plus metformin. *P < 0.05

Chronic pancreatitis is an inflammatory disease characterized by the atrophy of normal acini accompanied by obvious pancreatic fibrosis [22]. To investigate the role of metformin in eliminating chronic pancreatitis-induced fibrosis, pancreatic tissues from mice treated with vehicle, cerulein, or cerulein plus metformin were stained with Masson’s trichrome (Fig. 4a). As previously reported, the percentage of area stained with Masson’s trichrome was dramatically increased following cerulein treatment. Metformin decreased the Masson’s trichrome-stained area (Fig. 4b). PSCs are responsible for fibrosis. Under normal conditions, PSCs are maintained in a quiescent state. Once activated by external insults such as pancreatitis or pancreatic injury, PSCs become activated and express high levels of α-SMA, thus up-regulating the synthesis of extracellular matrix (ECM) [23]. Hence, we stained α-SMA to evaluate the activity of PSCs in different groups, and we found obvious effects of metformin on reducing the α-SMA positive area (Fig. 4c).

### Metformin impaired STAT3 signaling and inhibited proliferation

**Fig. 5:**
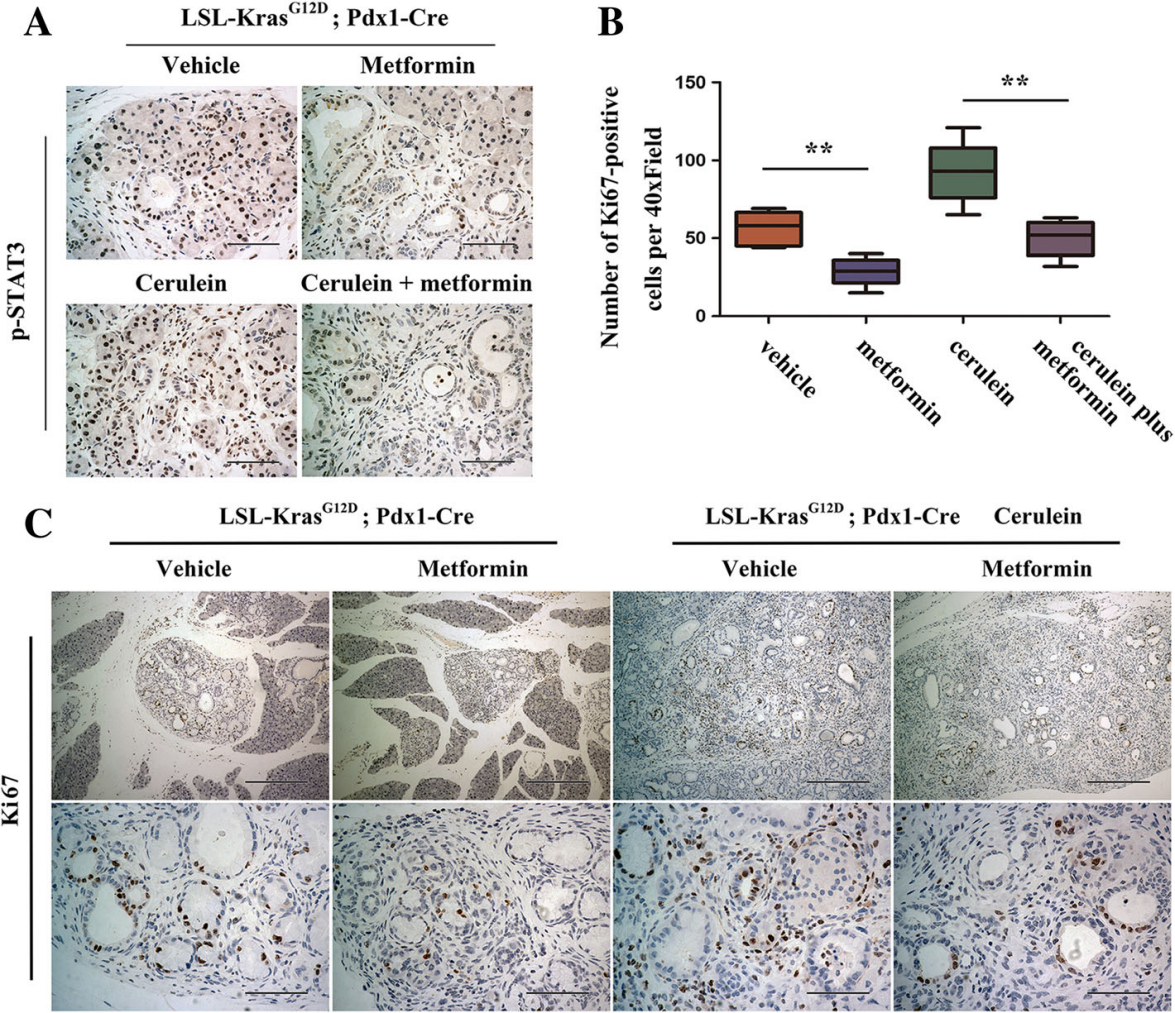
STAT3 signaling and cell proliferation were suppressed via metformin treatment. **a** Immunohistochemical staining of phospho-STAT3 in the pancreas of mice treated with vehicle, metformin, cerulein, and cerulein plus metformin. Scale bars =100 μm. **b** Quantification of the numbers of Ki67-positive cells in different groups. **P < 0.01. **c** Representative images stained by anti-Ki67. Scale bars: top row = 400 μm; 2nd row = 100 μm

A previous study has suggested a crucial role for STAT3 signaling in Kras-induced pancreatic tumorigenesis [8]. We evaluated the effect of metformin on the STAT3 pathways in a KC mouse model. Immunohistochemical (IHC) staining of p-STAT3 was detected in early mPanIN and the surrounding acini from KC mice treated with vehicle. Mice treated with metformin showed a decreased level of p-STAT3 staining (Fig. 5a). In cerulein-induced tissue of chronic pancreatitis, IHC staining of p-STAT3 was dramatically increased. However, metformin treatment impaired cerulein-induced p-STAT3 staining augmentation (Fig. 5a). Considering the evidence that STAT3 is involved in the proliferation of cancer-initiating cells as well as cancer cells [24, 25], we set out to investigate whether metformin impaired cell proliferation in precursor lesions. IHC staining of Ki67 was performed on the pancreas tissue of mice treated with vehicle or metformin. In mice that received metformin treatment, the pancreas showed a decreased number of Ki67-positive cells (Fig. 5b). Repeated cerulein injection induced a significant increase of Ki67 staining. However, it was reversed following metformin treatment (Fig. 5b). Collectively, our results revealed that metformin suppressed pancreatic oncogenesis in part through impairment of STAT3 signaling and inhibition of cell proliferation.

### Metformin reduced tumor burden and prolonged survival in KPC mice

**Fig. 6:**
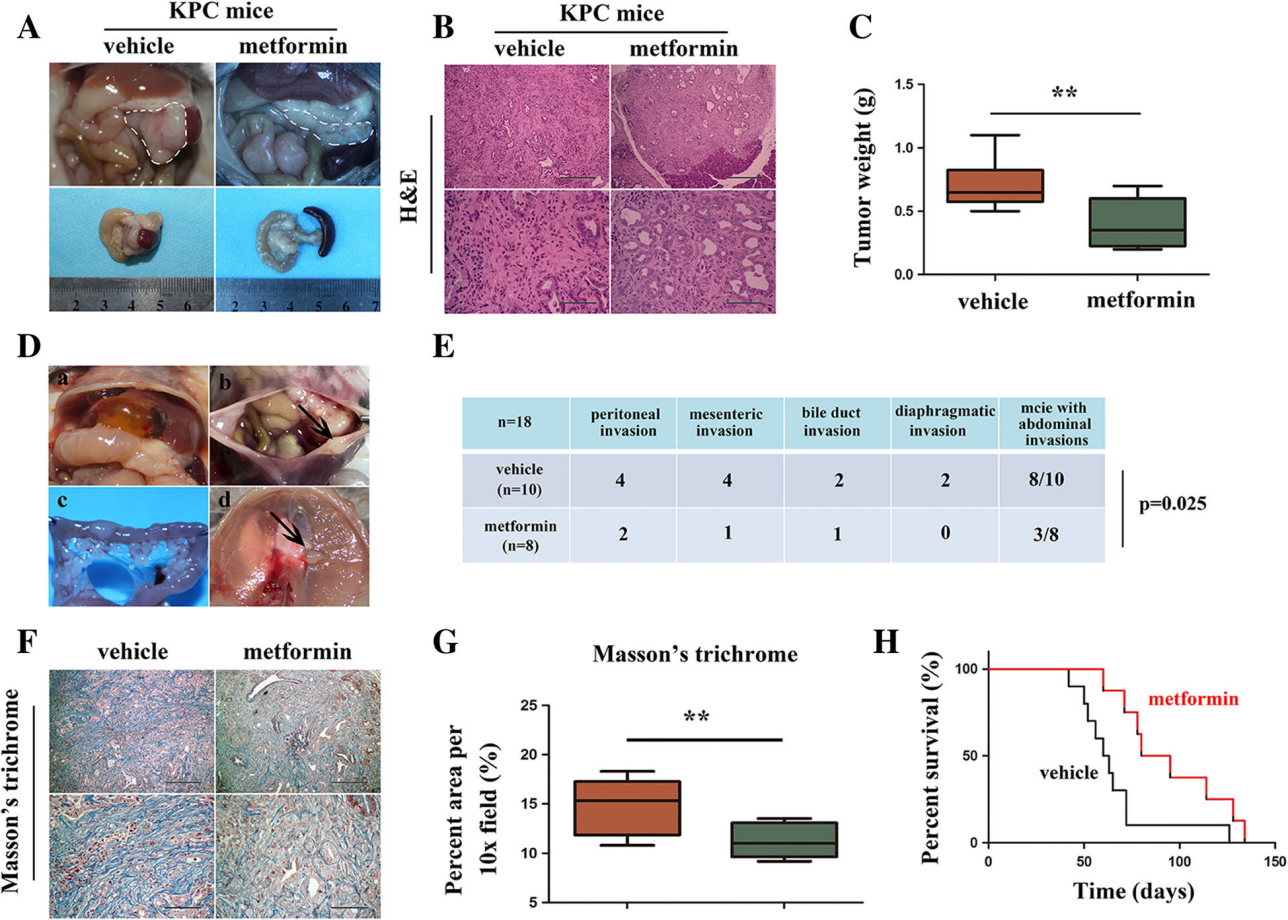
Metformin inhibited the growth and invasion of PDAC in a KPC mouse model. **a** Representative macroscopic images of PDAC in KPC mice treated with vehicle or metformin. The white dotted line shows the pancreas. **b** Histology of tumors from KPC mice in groups as indicated. **c** Quantification of tumor weight in mice treated with vehicle or metformin. **d** Representative images of bile duct invasion (**a**), peritoneal invasion and ascites (**b**), mesenteric invasion (**c**) and diaphragmatic invasion (**d**) identified in KPC mice. **e** Table listing the incidence of abdominal invasion in KPC mice treated as indicated. **f** Representative images of Masson’s trichrome staining. **g** Quantification of Masson’s trichrome in mice as indicated. **h** Kaplan-Meier survival analysis of KPC mice treated with vehicle or metformin. **P < 0.01

To further evaluate the therapeutic effect of metformin in pancreatic cancer treatment, we assessed whether treatment with metformin inhibited tumor progression in KPC mouse model. Starting at 6 weeks of age, KPC mice were treated daily with vehicle or metformin. As expected, KPC mice treated with metformin presented a prolonged overall survival (Fig. 6h). Accordingly, mice treated with metformin had a decreased tumor volume and tumor weight (Fig. 6a, c). Among those treated with vehicle, we observed abdominal invasions including peritoneal invasion (4/10), mesenteric invasion (4/10), diaphragmatic invasion (2/10) and bile duct invasion (2/10) (Fig. 6d, e). Metformin treatment dramatically decreased abdominal invasions, where just one out of eight showed mesenteric invasion (1/8), two showed peritoneal invasions (2/8), and one showed bile duct invasion (1/8) (Fig. 6e). Previous evidence suggested the role of metformin in pancreatic desmoplasia [5], as well as its role in reducing chronic pancreatitis-induced pancreatic fibrosis. We further evaluated its anti-fibrotic role in KPC mice. Similar to our previous report [15], the Masson’s trichrome-stained area was decreased following metformin treatment (Fig. 6f, g). In addition, we detected activation of AMPK signaling, demonstrated as increased expression of p-AMPK and p-ACC (Additional file 5: Fig. S4A-B). We also found that metformin treatment down-regulated the levels of p-mTOR and p-P70S6K (Additional file 5: Fig. S4A-B). Collectively, these data suggest that metformin treatment decreased tumor burden and abdominal invasion. Importantly, it prolonged the overall survival of KPC mice.

## Discussion

The development of genetically engineered mouse models has led to an understanding of the initiation and progression of pancreatic cancer, thus providing a more efficient tool for the research of pancreatic cancer prevention and treatment [20]. Oncogenic Kras-mediated PDAC mouse models recapitulate tumor onset and progression from ADM to mPanINs and eventually to invasive pancreatic cancer. We found that intake of metformin delayed pancreatic tumorigenesis in KC mouse model, represented by a decreased percentage of early lesions (ADM and mPanIN1) and late mPanIN lesions (mPanIN2 and mPanIN3). Furthermore, metformin suppressed chronic pancreatitis-induced tumorigenesis, and it showed a promising effect in reducing chronic pancreatitis-induced pancreatic desmoplastic reaction. Accordingly, the activity of STAT3 signaling was decreased in KC mice as well as mice with chronic pancreatitis following metformin treatment. More importantly, metformin induced tumor regression and prolonged the overall survival of KPC mice.

Accumulating evidence has suggested that metformin has a cancer preventive effect [26, 27]. Patients who received metformin demonstrated a decreased risk of incident cancer, including ovarian cancer [28], prostate cancer [29], and colorectal cancer [26]. A previous study indicated that metformin inhibited cancer cell proliferation and stemness in non-small cell lung cancer (NSCLC) [30], and it suppressed tobacco carcinogen-induced lung tumorigenesis. A subsequent study showed that metformin’s anti-tumorigenic effects could be mediated by inhibiting the phosphorylation of insulin-like growth factor-I receptor/insulin receptor (IGF-1R/IR), AKT, ERK, and mTOR [31]. PDAC is believed to initiate from precursor lesions of the pancreas such as ADM and PanINs, which could be induced by oncogenic Kras or pancreatitis [32]. Our data support the idea that, in accordance with its cancer preventive effect in other cancers, metformin plays an important role in preventing pancreatic tumorigenesis.

Chronic pancreatitis has been accepted as one of the most important risk factors for PDAC [16, 33]. A previous study suggested that on the background of oncogenic Kras, chronic pancreatitis is essential for the initiation and acceleration of PDAC [16]. Recent studies suggested that the interleukin 17 pathway mediates the pancreatitis-to-cancer transition and induces the activation of the JAK2-STAT3 pathway during ADM and in early PanIN lesions [34]. Chronic pancreatitis can also contribute to the initiation and progression of PDAC by abrogating the senescence barrier characteristic of low-grade mPanINs. Suppression of pancreatitis promoted tissue repair and retarded PanIN expansion [35]. Our data shows that, in line with previous findings, mice treated with cerulein induced chronic pancreatitis, which presented an almost complete replacement of normal pancreatic tissue with ductal architecture and deposition of a large amount of collagen and fibril in the stroma. The majority of acini were replaced by PanIN lesions and metaplasia. Surprisingly, we found that metformin significantly retarded the chronic pancreatitis to PDAC transition and reduced pancreatic fibrosis. Accordingly, the pancreatic proliferation index, measured by Ki67, was also diminished.

Cancer initiation is associated with abnormal alteration of several signaling pathways, among which the signal transducer and activator of transcription (STAT) proteins are included [8]. STAT3 is present in the cytoplasm under basal conditions. Once activated, STAT3 dimerizes and localizes to the nucleus [36]. Previous studies have revealed that STAT3 is persistently activated in a wide range of human malignancies, and it exerts diverse roles in cancer cell proliferation, epithelial to mesenchymal transition (EMT), invasion and metastasis [37]. In addition, STAT3 promotes cancer development by promoting the self-renewal and differentiation of cancer stem cells (CSCs), which play crucial roles in tumorigenesis [38]. STAT3 has been identified as a key regulator in epithelial and gastric carcinogenesis [39, 40]. In pancreatic cancer, STAT3 was observed during all stages of pancreatic oncogenesis, and inhibition or loss of STAT3 reduced oncogenic KRAS-induced ADM and PanIN formation [8]. Accordingly, we showed expression of p-STAT3 in precursor lesions from KC mice, and chronic pancreatitis induced an increased STAT3 activity. Treatment with metformin reduced the activation of STAT3 signaling in KC mice and mice with chronic pancreatitis.

For pancreatic cancer, gemcitabine remains the mainstay of chemotherapy [41]. S-1 is also applied for adjuvant chemotherapy for resected pancreatic cancer [3]. Previous evidence showed that patients with pancreatic cancer benefit from the FOLFIRINOX scheme and nab-paclitaxel [42, 43]. For those who present with metastatic pancreatic cancer and have received gemcitabine-based therapy previously, nanoliposomal irinotecan in combination with fluorouracil and folinic acid extends patients’ median overall survival [44]. However, therapeutic efficiency was achieved at the cost of a high incidence of adverse events such as leucopenia, neutropenia, liver injury or gastrointestinal discomfort. Numerous studies have revealed the therapeutic effects of metformin in diverse cancer types including endometrial cancer [45], castration-resistant prostate cancer [46], and breast cancer [47]. We previously reported that metformin inhibited tumor growth in subcutaneous and orthotopic models of pancreatic cancer [15]. Recent study revealed that mitochondrially targeted metformin (MitoMet) considerably more efficiently killed pancreatic cancer cells and suppressed pancreatic tumors in vivo by targeting the mitochondrial complex I (CI) [48]. Here, we find that in a genetically engineered mouse model (KPC mice), metformin also showed therapeutic efficiency with a decreased tumor burden and lower incidence of abdominal invasion. More importantly, metformin prolongs the overall survival of KPC mice. Desmoplastic reaction is one of the characteristics of PDAC. PSCs are responsible for pancreatic fibrosis, during which PSCs transform from a quiescent state into α-SMA positive activated state [49]. Substantial evidence has revealed that fibrotic stroma establish a fertile microenvironment for tumor growth and distant metastasis [50, 51]. We found that in KPC mouse model, treatment with metformin inhibited the activation of PSCs and suppressed fibrosis in PDAC tissues.

## Conclusion

The present study showed that in genetically engineered mouse models of PDAC, metformin inhibits oncogenic Kras-induced pancreatic tumorigenesis. Treatment with metformin suppressed chronic pancreatitis-induced metaplasia and the pancreatitis-to-cancer transition, accompanied with the elimination of fibrosis. Additionally, metformin reduced tumor burden and prolonged the overall survival.

## Additional files

Additional file 1: Figure S2. Gene targeting strategy for the generation and breeding of KC (A) and KPC mice (B). (TIFF 308 kb)

Additional file 2: Figure S3. Polymerase chain reaction (PCR) showing the genotyping of Kras^G12D^ (A), P53 (B) and Cre recombinase (C). (TIFF 8808 kb)

Additional file 3: Table S1. Primer sequences used for the genotyping of transgenic mice (DOC 27 kb)

Additional file 4: Figure S1. The kinetics of tumor formation in KC and KPC mice. (A) HE staining of the pancreatic tissues from KC mice which were sacrificed at different time points (6, 10, 14, 18 weeks). (B) The macroscopic images and HE staining showing the pancreas from KPC mice which were sacrificed at different time points (3, 6, 9, 12 weeks). (TIFF 10685 kb)

Additional file 5: Figure S4. The effect of metfomin on AMPK/mTOR signaling. (A) Immunohistochemical staining of p-AMPK and p-mTOR in pancreatic tissues from KPC mice treated with vehicle or metformin. (B) Western blotting assays show the expression of p-AMPK, AMPK, p-mTOR, mTOR, p-P70S6K, and P70S6K in pancreatic tissues from KPC mice treated as indicated. (TIFF 9891 kb)

## Abbreviations

ADM: Acinar-to-ductal metaplasia
CSCs: Cancer stem cells
ECM: Extracellular matrix
EGFR: Epidermal growth factor receptor
EMT: Epithelial to mesenchymal transition
KC: Kras^LSL-G12D/+^; Pdx1-Cre
KPC: Kras^LSL-G12D/+^; Trp53^fl/+^; Pdx1-Cre
MPanIN: Mouse pancreatic intraepithelial neoplasia
PDAC: Pancreatic ductal adenocarcinoma
PSCs: Pancreatic stellate cells
STAT3: Signal transducer and activator of transcription 3
