# Supplementary figures and images for: Metformin suppresses cancer initiation and progression in genetic mouse models of pancreatic cancer
Source: Mol Cancer. 2017 Jul 24;16:131. doi: 10.1186/s12943-017-0701-0 (PMC5525317; doi:10.1186/s12943-017-0701-0)

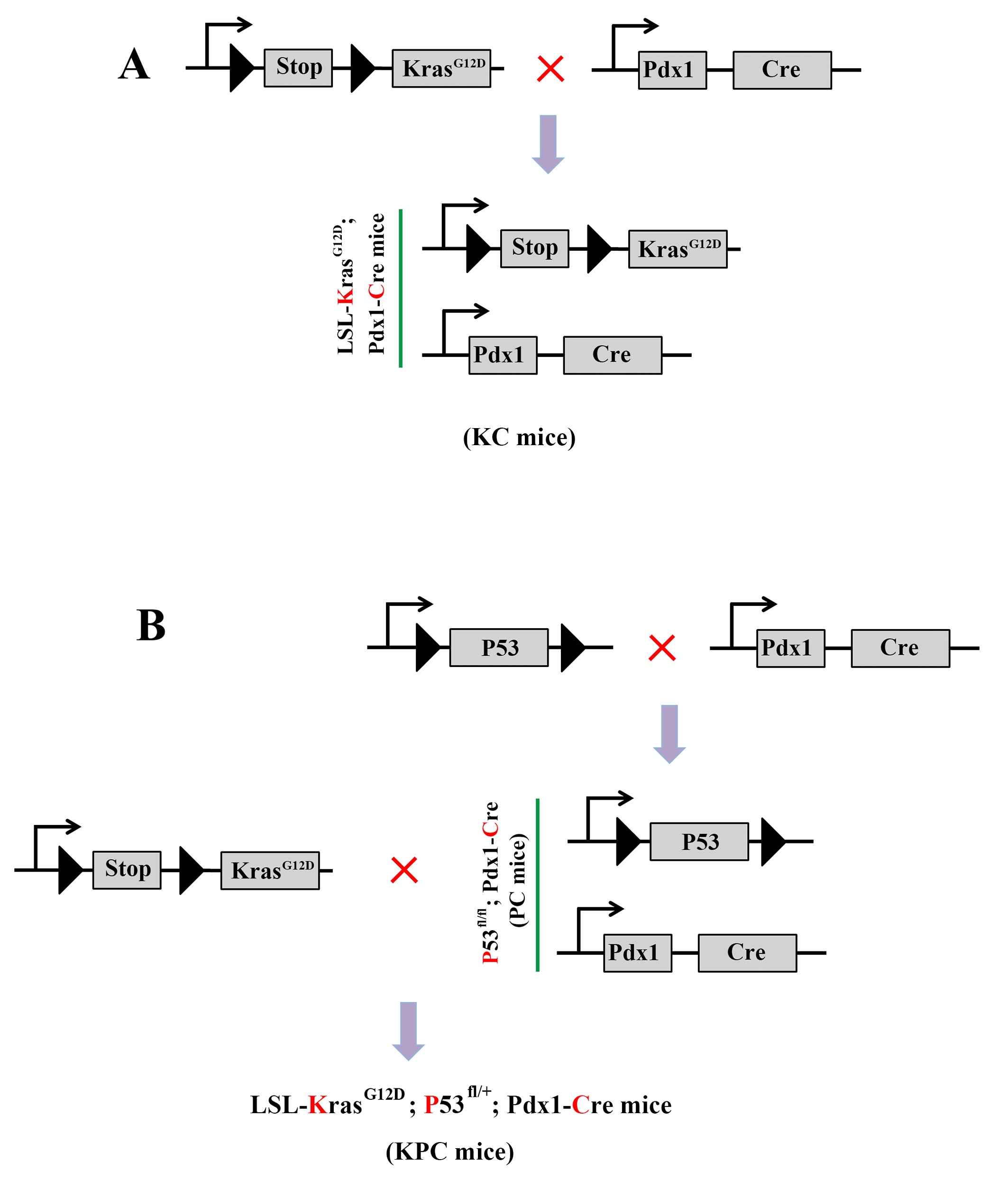

Supplement: Supplementary file 1 — Gene targeting strategy for the generation and breeding of KC (A) and KPC mice (B). (TIFF 308 kb) [file 12943_2017_701_MOESM1_ESM.tif]

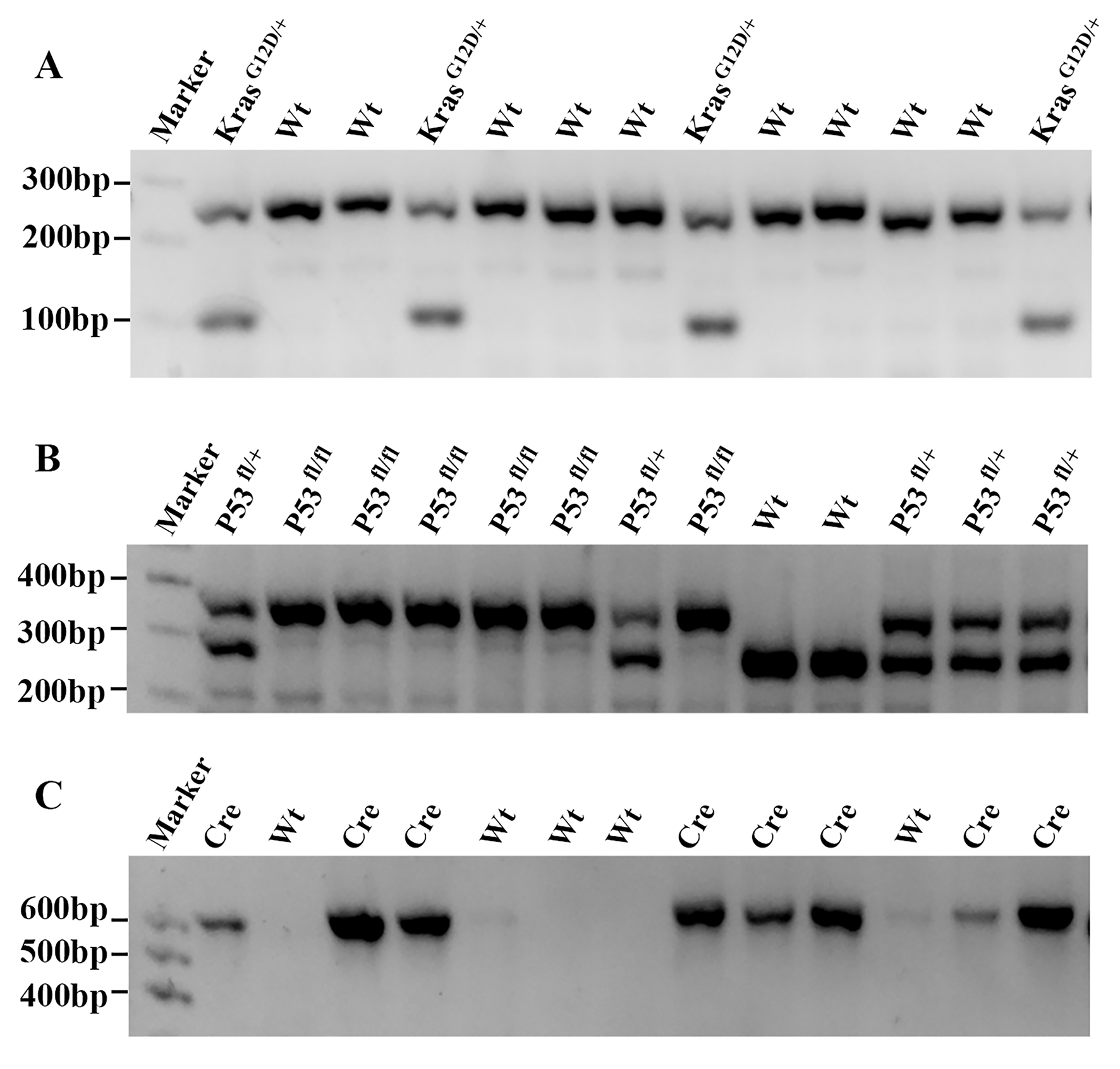

Supplement: Supplementary file 2 — Polymerase chain reaction (PCR) showing the genotyping of KrasG12D (A), P53 (B) and Cre recombinase (C). (TIFF 8808 kb) [file 12943_2017_701_MOESM2_ESM.tif]

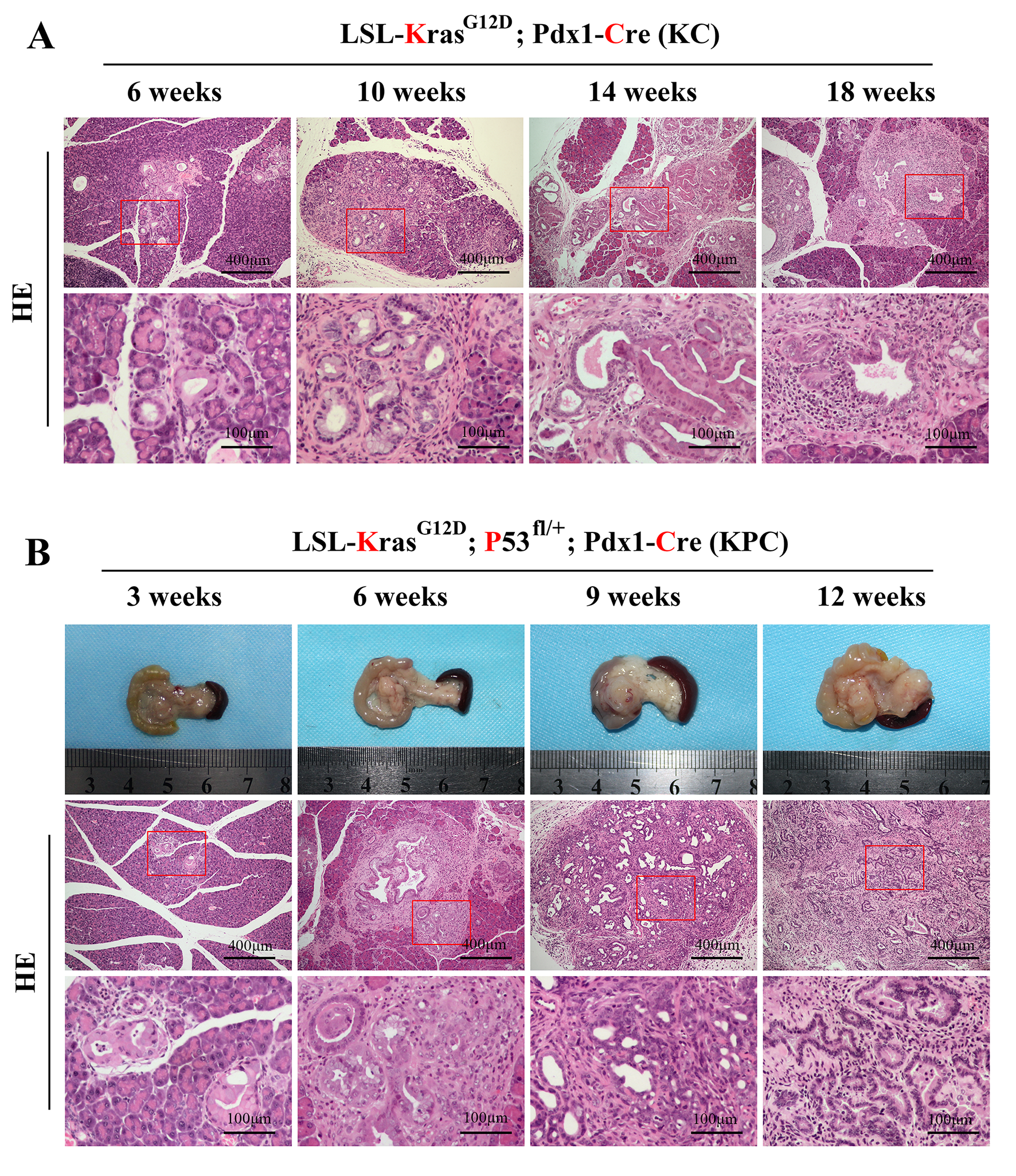

Supplement: Supplementary file 4 — The kinetics of tumor formation in KC and KPC mice. (A) HE staining of the pancreatic tissues from KC mice which were sacrificed at different time points (6, 10, 14, 18 weeks). (B) The macroscopic images and HE staining showing the pancreas from KPC mice which were sacrificed at different time points (3, 6, 9, 12 weeks). (TIFF 10685 kb) [file 12943_2017_701_MOESM4_ESM.tif]

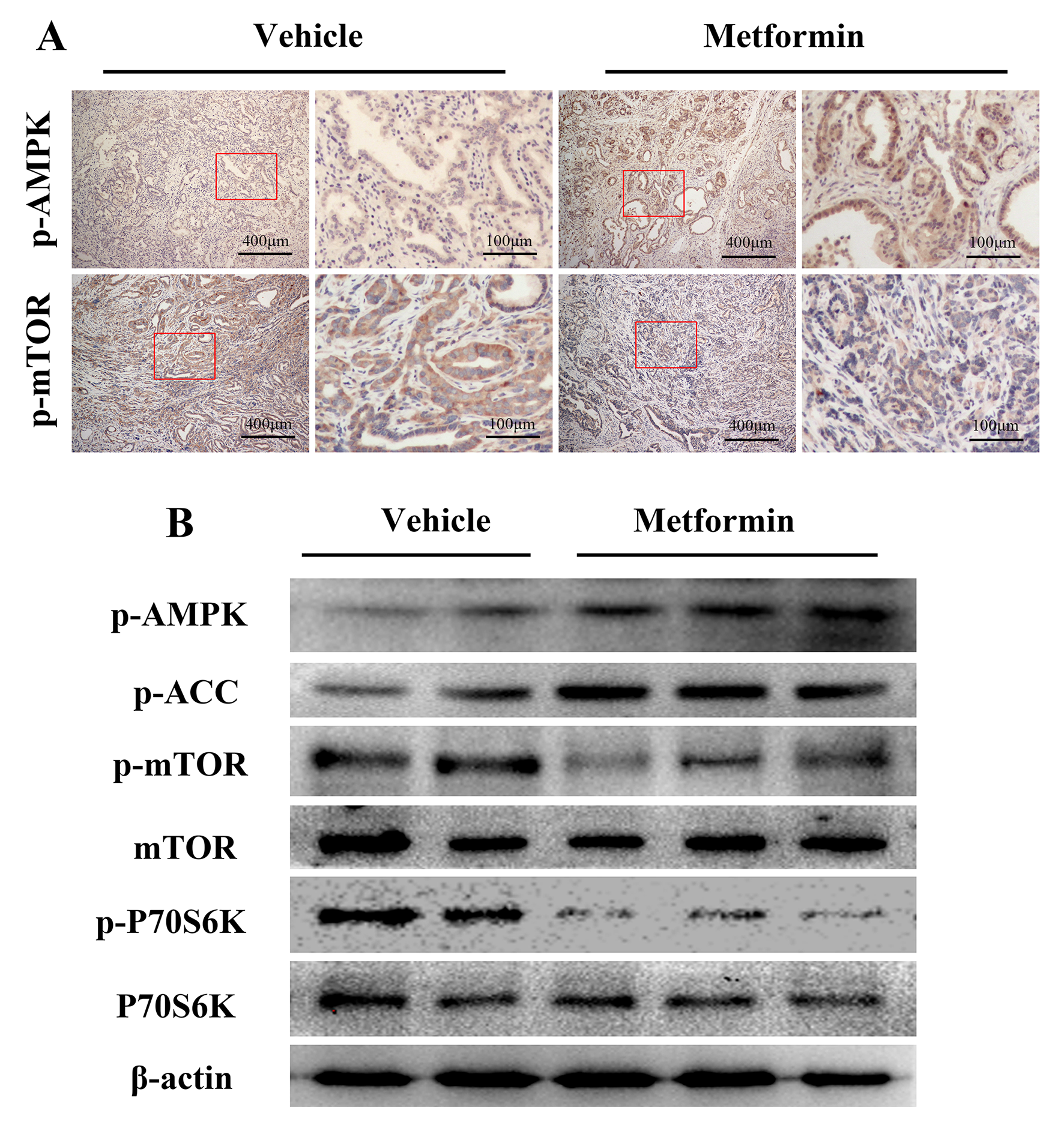

Supplement: Supplementary file 5 — The effect of metfomin on AMPK/mTOR signaling. (A) Immunohistochemical staining of p-AMPK and p-mTOR in pancreatic tissues from KPC mice treated with vehicle or metformin. (B) Western blotting assays show the expression of p-AMPK, AMPK, p-mTOR, mTOR, p-P70S6K, and P70S6K in pancreatic tissues from KPC mice treated as indicated. (TIFF 9891 kb) [file 12943_2017_701_MOESM5_ESM.tif]
