# Supplementary material for: Metformin suppresses cancer initiation and progression in genetic mouse models of pancreatic cancer
Source: Mol Cancer. 2017 Jul 24;16:131. doi: 10.1186/s12943-017-0701-0 (PMC5525317; doi:10.1186/s12943-017-0701-0)
Supplement: Supplementary file 3 — Primer sequences used for the genotyping of transgenic mice (DOC 27 kb) [file 12943_2017_701_MOESM3_ESM.doc]

**Additional file 3: Table S1**: Primer sequences used for the genotyping of transgenic mice.

| **Genes** | **Primer Sequences** |
| --- | --- |
| LSL-Kras^G12D^ | 5’-TGTCTTTCCCCAGCACAGT-3’  5’-CTGCATAGTACGCTATACCCTGT-3’  5’-GCAGGTCGAGGGACCTAATA -3’ |
| P53^fl/fl^ or P53^fl/+^ | 5’-CACAAAAACAGGTTAAACCCAG -3’  5’-AGCACATAGGAGGCAGAGAC -3’ |
| Pdx1-Cre | 5’-CTGGACTACATCTTGAGTTGC -3’  5’-GGTGTACGGTCAGTAAATTTG -3’ |
